# Refractory Hypereosinophilic Syndrome With Clonal T‐Cell Receptor Rearrangement Treated With JAK Inhibition Following Incomplete Response to IL‐5 Blockade

**DOI:** 10.1155/crh/9477679

**Published:** 2026-09-04

**Authors:** Kevin Wayne McKaughan, Phu V. Truong, Gregory Bongers

**Affiliations:** ^1^ The University of Kansas School of Medicine–Wichita, Wichita, Kansas, USA; ^2^ Cancer Center of Kansas, Wichita, Kansas, USA, cancercenterofkansas.com; ^3^ Family MedCenter, Derby, Kansas, USA

## Abstract

Hypereosinophilic syndrome (HES) comprises a heterogeneous group of disorders characterized by persistent eosinophilia and eosinophil‐mediated organ involvement. Although interleukin‐5 (IL‐5) inhibition is an established treatment strategy, some patients experience persistent or recurrent disease despite therapy. Janus kinase (JAK) inhibition has emerged as a potential therapeutic option in refractory cases. We report a 63‐year‐old White male who presented with a diffuse pruritic erythematous rash, marked leukocytosis with eosinophils comprising approximately 83% of circulating leukocytes, and markedly elevated serum IgE levels. Bone marrow biopsy demonstrated a hypercellular marrow (approximately 89%) with eosinophils accounting for approximately 75% of nucleated cells. Extensive evaluation, including cytogenetic, fluorescence in situ hybridization, and molecular testing for myeloid neoplasms, was unrevealing, while peripheral blood polymerase chain reaction demonstrated a clonal T‐cell receptor gene rearrangement. Despite prior hydroxyurea therapy, prolonged treatment with mepolizumab, and repeated corticosteroid courses, the patient developed recurrent disease with an absolute eosinophil count of 2760/μL. Ruxolitinib was initiated while IL‐5 inhibition was continued. Following JAK inhibition, leukocyte and eosinophil counts normalized, corticosteroids were discontinued, and disease control was maintained at 3‐month follow‐up. This case highlights the potential role of JAK inhibition as adjunctive therapy in refractory HES with clonal T‐cell receptor rearrangement. Further studies are needed to define the safety, durability, and optimal integration of JAK inhibition in patients with refractory eosinophilic disorders.

## 1. Introduction

Hypereosinophilic syndrome (HES) is defined as persistent eosinophilia ≥ 1.5 × 10^9^/L on at least two occasions separated by 4 weeks, or evidence of marked tissue eosinophilic infiltration after exclusion of secondary causes [1]. HES comprises a heterogeneous group of disorders, including myeloid, lymphocytic, and idiopathic variants, reflecting distinct pathogenic mechanisms. Lymphocytic forms of HES are thought to arise from dysregulated T‐cell populations that promote eosinophil expansion through cytokine production, most notably interleukin‐5 (IL‐5) [2].

Targeted IL‐5 inhibition with mepolizumab has significantly improved disease control in many patients with HES, reducing flare frequency in clinical trials [3]. However, incomplete responses and persistent corticosteroid requirements remain therapeutic challenges in a subset of patients. Other immunomodulatory therapies, including interferon‐α, have also demonstrated efficacy in selected patients with lymphocytic and steroid‐refractory disease [2]. Janus kinase (JAK) inhibition has emerged as a potential therapeutic strategy in refractory eosinophilic disorders because it modulates multiple cytokine signaling pathways involved in eosinophil proliferation and activation [4]. Although clinical experience remains limited, ruxolitinib has been reported to improve disease activity in patients with CD3−CD4+ lymphocytic‐variant HES [5].

We describe a patient with refractory HES and a clonal T‐cell receptor rearrangement who demonstrated incomplete response to IL‐5 inhibition and subsequently achieved disease control following addition of ruxolitinib.

## 2. Case Presentation

A 63‐year‐old White male presented in May 2024 for evaluation of severe eosinophilia and a diffuse pruritic erythematous rash involving the trunk and extremities. Initial laboratory evaluation revealed marked leukocytosis (white blood cell count 88.5 × 10^3^/μL) with eosinophils comprising approximately 83% of circulating leukocytes, mild anemia, elevated lactate dehydrogenase, markedly elevated serum IgE (9060 IU/mL), elevated IgM (465 mg/dL), elevated IgG (2638 mg/dL), and low‐normal IgA (64 mg/dL). Selected laboratory values across key phases of the disease course are summarized in Table 1. A comprehensive evaluation was undertaken to exclude secondary, infectious, autoimmune, and clonal etiologies, consistent with established diagnostic approaches to hypereosinophilia [2].

**TABLE 1:** Selected laboratory values during key phases of disease.

Disease phase	Date	WBC (× 10^3^/μL)	ALC (/μL)	AEC (/μL)	Hgb (g/dL)	IgE (IU/mL)	Treatment
Presentation	05/06/24	88.5	—	78,960	12.5	9060	None
Hydroxyurea response	07/22/24	19.0	2.28	—	10.6	—	Hydroxyurea, 20 mg Prednisone
Peak IgE during mepolizumab	08/12/24	4.90	0.88	—	11.9	46,891	Mepolizumab, 20 mg Prednisone
Relapse on mepolizumab	09/20/24	25.7	0.51	18,000	11.6	—	Mepolizumab, 20 mg Prednisone
Hematologic nadir	11/22/24	6.80	1.12	2600	7.8	—	Mepolizumab, 20 mg Prednisone
Preruxolitinib relapse	09/30/25	11.5	0.69	2760	9.7	16,322	Mepolizumab, 40 mg Prednisone
Postruxolitinib	12/23/25	5.90	1.65	Normalized	11.0	—	Mepolizumab, Ruxolitinib
Most recent follow‐up	01/20/26	4.20	1.00	Normalized	11.0	16,323	Mepolizumab, Ruxolitinib

*Note:* WBC, white blood cell count. Normalized indicates eosinophil counts within the institutional reference range.

Abbreviations: AEC, absolute eosinophil count; ALC, absolute lymphocyte count.

**FIGURE 1:**
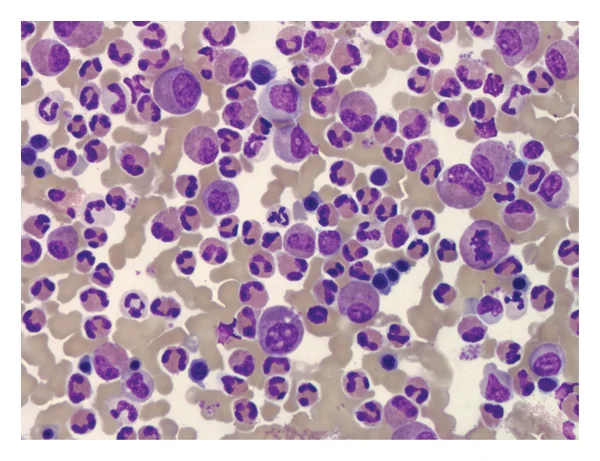
Bone marrow aspirate smear obtained at presentation demonstrating numerous eosinophilic precursors and mature eosinophils, consistent with marked marrow eosinophilia. Bone marrow biopsy revealed a hypercellular marrow (approximately 89%) with eosinophils comprising approximately 75% of nucleated cells.

Bone marrow biopsy demonstrated a hypercellular marrow (approximately 89%) with eosinophils accounting for approximately 75% of nucleated cells, marked expansion of the myeloid compartment, mild reticulin fibrosis, and no increase in blasts (Figure 1). Bone marrow flow cytometry demonstrated marked eosinophilia without evidence of a diagnostically significant abnormal hematolymphoid population. Cytogenetic and molecular testing was unrevealing, including normal conventional cytogenetics and negative testing for JAK2, CALR, MPL, BCR‐ABL1, PDGFRA, PDGFRB, FGFR1, KIT D816V, and a broad myeloid next‐generation sequencing panel. Peripheral blood polymerase chain reaction demonstrated a clonal T‐cell receptor gamma gene rearrangement, which was subsequently confirmed on repeat testing performed during tertiary evaluation.

Given the degree of eosinophilia and concern for eosinophil‐mediated end‐organ damage, a comprehensive evaluation was undertaken. Echocardiography and cardiac magnetic resonance imaging demonstrated preserved left ventricular systolic function without evidence of eosinophilic cardiac involvement. PET/CT demonstrated diffuse hypermetabolic lymphadenopathy involving cervical, axillary, mediastinal, and hilar nodal stations, as well as hypermetabolic pulmonary nodules. Endobronchial ultrasound‐guided fine needle aspiration of mediastinal lymph node stations 4 R/7 and 11R demonstrated abundant eosinophils in the background without evidence of carcinoma. Concurrent flow cytometric analysis demonstrated no evidence of a B‐cell or T‐cell lymphoproliferative disorder. Subsequent mediastinoscopy with excisional lymph node biopsy demonstrated no evidence of lymphoma or other lymphoproliferative disorder.

Hydroxyurea 500 mg twice daily was initiated with partial hematologic improvement. In July 2024, hydroxyurea was discontinued, and mepolizumab 300 mg subcutaneously every 4 weeks was initiated. Although initial clinical improvement was observed, the patient experienced recurrent disease activity over subsequent months requiring repeated corticosteroid courses, including prednisone doses of up to 40 mg twice daily despite ongoing IL‐5 inhibition.

In February 2025, tertiary evaluation at Mayo Clinic favored a lymphocytic etiology of HES and recommended ruxolitinib 20 mg twice daily. Insurance approval was delayed, and mepolizumab was continued. In September 2025, recurrent disease was documented with an absolute eosinophil count of 2760/μL despite ongoing IL‐5 inhibition and corticosteroid therapy. Ruxolitinib was initiated in October 2025 while mepolizumab was continued.

**FIGURE 2:**
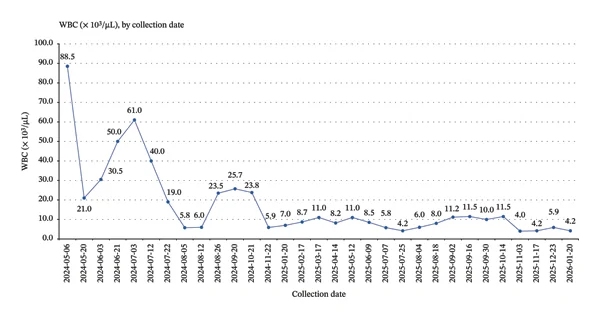
White blood cell count throughout the disease course demonstrating marked leukocytosis at presentation, partial response to hydroxyurea, subsequent control with mepolizumab, and sustained normalization following initiation of ruxolitinib.

**FIGURE 3:**
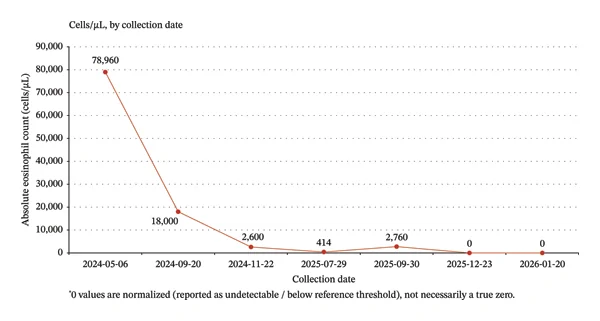
Absolute eosinophil count during treatment course. Despite the initial response to therapy, recurrent eosinophilia developed while receiving mepolizumab and corticosteroids. Following the addition of ruxolitinib, eosinophil counts normalized and remained controlled.

Following initiation of ruxolitinib, leukocyte and eosinophil counts normalized (Figures 2 and 3). Systemic corticosteroids were tapered and discontinued. Disease control was maintained at 3‐month follow‐up. Serum IgE peaked at 46,891 IU/mL in August 2024 and remained elevated at 16,322–16,323 IU/mL through September 2025 and January 2026. At the most recent follow‐up, approximately 20 months after the initial presentation, the patient remained on ruxolitinib 10 mg twice daily in combination with mepolizumab 300 mg monthly with sustained disease control and no significant adverse effects.

## 3. Discussion

HES comprises a heterogeneous group of disorders with diverse pathogenic mechanisms. Lymphocytic forms of HES are characterized by cytokine‐driven eosinophil expansion, most commonly mediated by IL‐5 produced by aberrant T‐cell populations, resulting in reactive eosinophilia rather than primary clonal eosinophil proliferation [2]. Our patient demonstrated a clonal T‐cell receptor gamma gene rearrangement and several clinical features frequently associated with lymphocytic disease, including diffuse pruritic rash, markedly elevated serum IgE levels (peaking at 46,891 IU/mL), elevated IgM levels, diffuse lymphadenopathy, and recurrent eosinophilia. However, definitive classification as lymphocytic‐variant HES was limited by the absence of available flow cytometric evidence demonstrating an aberrant T‐cell immunophenotype. Prior consensus criteria have defined lymphocytic‐variant HES as a distinct HES subtype characterized by aberrant T‐cell immunophenotypes, including CD3−CD4^+^ populations, with clonality serving as a supporting feature rather than the sole diagnostic criterion [1].

IL‐5 plays a central role in eosinophil growth and survival. However, IL‐5 is not the sole eosinophilopoietic cytokine; interleukin‐3 (IL‐3) and granulocyte‐macrophage colony‐stimulating factor (GM‐CSF) also contribute to eosinophil expansion and activation [1]. These cytokines signal through downstream JAK‐STAT pathways [4], providing a biologic rationale for JAK inhibition as a means of attenuating signaling from multiple eosinophilopoietic cytokines simultaneously. This may be particularly relevant in patients with eosinophilic disorders who experience persistent disease despite IL‐5 blockade, suggesting continued activity of alternative cytokine pathways.

Consistent with this mechanistic rationale, ruxolitinib has been reported to improve disease control in CD3−CD4+ lymphocytic‐variant HES [5]. In these patients, the aberrant CD3−CD4+ T‐cell population persisted despite therapy and may have contributed to eosinophil relapse following treatment discontinuation. These observations suggest that JAK inhibition functions as a suppressive rather than curative strategy, requiring ongoing pathway inhibition to maintain disease control.

In the present case, addition of ruxolitinib to ongoing mepolizumab therapy was associated with normalization of eosinophil counts and sustained clinical improvement after incomplete response to hydroxyurea, maximal IL‐5 blockade, and repeated corticosteroid courses.

This report is limited by its single‐patient design. Attribution of clinical response solely to ruxolitinib is not possible because mepolizumab therapy was continued and corticosteroids overlapped with treatment initiation. Additionally, a skin biopsy was not performed, precluding histologic evaluation of the cutaneous eruption and exclusion of cutaneous T‐cell lymphoma. Although a clonal T‐cell receptor rearrangement was identified, flow cytometric evidence of an aberrant T‐cell population was unavailable, limiting definitive classification as lymphocytic‐variant HES. Serial cytokine measurements were not obtained, precluding direct mechanistic assessment. Long‐term durability beyond current follow‐up remains uncertain.

## 4. Conclusion

HES with clonal T‐cell receptor rearrangement may remain active despite targeted IL‐5 inhibition. In this case, addition of JAK inhibition to ongoing IL‐5 blockade was associated with sustained disease control and normalization of eosinophil counts. Further prospective investigation is warranted to define the safety, durability, and optimal integration of JAK inhibition in refractory eosinophilic disorders, particularly those with features suggestive of a lymphocytic disease process.

## Author Contributions

Kevin Wayne McKaughan contributed to literature review, manuscript preparation, data collection, and manuscript revision. Phu V. Truong contributed to patient care, case conceptualization, manuscript review, and supervision. Gregory Bongers contributed to patient care and manuscript review.

## Funding

The authors received no financial support for the research, authorship, and/or publication of this article.

## Disclosure

All authors reviewed and approved the final manuscript.

## Consent

Written informed consent was obtained from the patient for publication of this case report and any accompanying images.

## Conflicts of Interest

The authors declare no conflicts of interest.

## Data Availability

All data generated or analyzed during this study are included in this published article.
